# Economic overview of falls among elderly Brazilians

**DOI:** 10.1590/S2237-96222022000200024

**Published:** 2022-09-09

**Authors:** Andressa Pinheiro, Ariadne Machado Schmidt, Camila Simon, Conceição de Maria Aguiar Barros Moura, Diane Guerra, Geise dos Santos Klipel, Gustavo Rodrigues Oliveira, Mariana Ramalho Rodrigues, Maria Letícia Rodrigues Ikeda, Nêmora Tregnago Barcellos

**Affiliations:** 1 Universidade do Vale do Rio do Sinos, São Leopoldo, RS, Brazil

Dear Editors,

We congratulate the authors of the article ‘Costs of Hospital Admission Authorizations due to falls among older people within the Brazilian National Health System, Brazil, 2000-2020: a descriptive study’, developed by Lima et al.^1^ and published in Issue No. 1, Volume 31 of this journal. It is a very well written and pleasant to read article and, despite being a macro or top-down study, therefore having limitations regarding accuracy with regard to health technologies, it allows us to visualize a panorama of the costs of falls among the Brazilian elderly.

We examined the study based on the evaluation tool proposed by Silva et al.,^2^ described in their article ‘Economic evaluation of health technologies: checklist for critical analysis of published articles’. The methodological quality of the study is so clear that we consider that it meets most of evaluation criteria proposed in the checklist that are pertinent to this type of study.

We would like to suggest that, although total costs were adjusted according to the Broad National Consumer Price Index (Índice Nacional de Preços ao Consumidor Amplo - IPCA), we believe that they could have been converted to US dollars, as this is a less volatile currency than the Brazilian real (BRL). Moreover, the authors could have used methods to extrapolate the results over the long term, thus giving us a perspective of the problem of falls in the future.

Reading the article led us to reflect on the role of Primary Health Care in reducing the costs of falls among the elderly, given the existence of programs such as Home Care and Pharmaceutical Care, which can act to reduce hospitalization time and prevent falls. We suggest that, in a future study, the role of these initiatives in reducing financial impacts related to falls should be addressed.

Based on the results indicated in the article, we agree with the authors when they conclude that falls are, for the most part, avoidable events. Public health service managers, health workers and society in general should be more attentive to these events and to the conditions of the elderly population, developing strategies and prevention measures, not only for the direct costs pointed out by the study, but also for the non-medical, indirect and intangible costs that arise from these health conditions.
